# Supplementary figures and images for: Reward foraging task and model-based analysis reveal how fruit flies learn value of available options
Source: PLoS One. 2020 Oct 2;15(10):e0239616. doi: 10.1371/journal.pone.0239616 (PMC7531776; doi:10.1371/journal.pone.0239616)

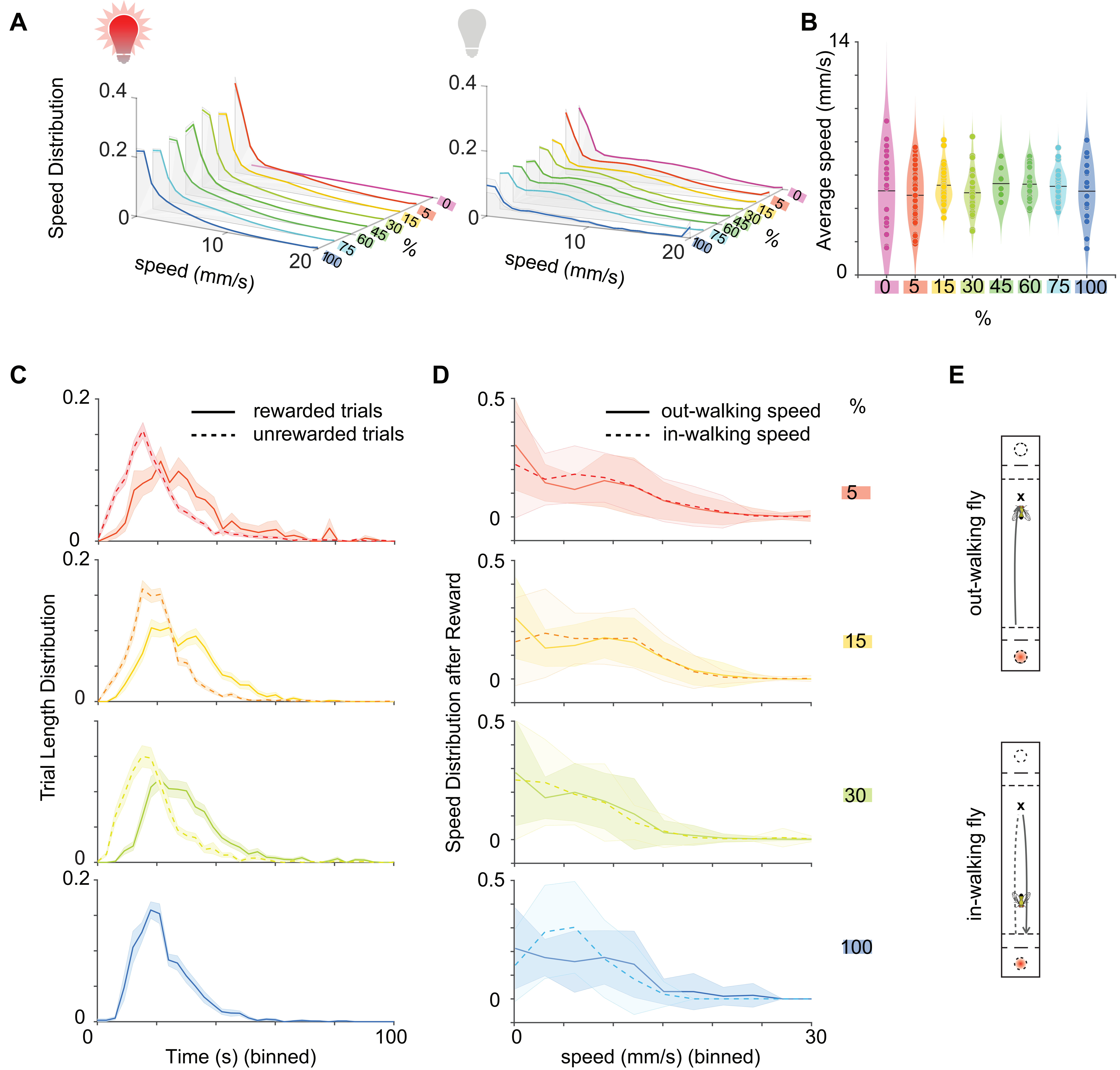

Supplement: S1 Fig — A Speed distribution of fly populations. Left: stimulated trials (grey light bulb). Right: unstimulated trials (red light bulb). B Average walking speed per condition. C Trial length distribution of 5,15,30 and 100% condition populations. Solid lines show rewarded trials (longer) and dashed lines show unrewarded trials (shorter). D Speed distribution after a reward. Solid lines: out of the reward zone walking speed. Dashed lines: in-walking speed when returning from walking out (same trial as out-walking speed). In-walking is on average slower than out-walking. E Pictogram of out-walking and in-walking traces. (PDF) [file pone.0239616.s001.pdf]

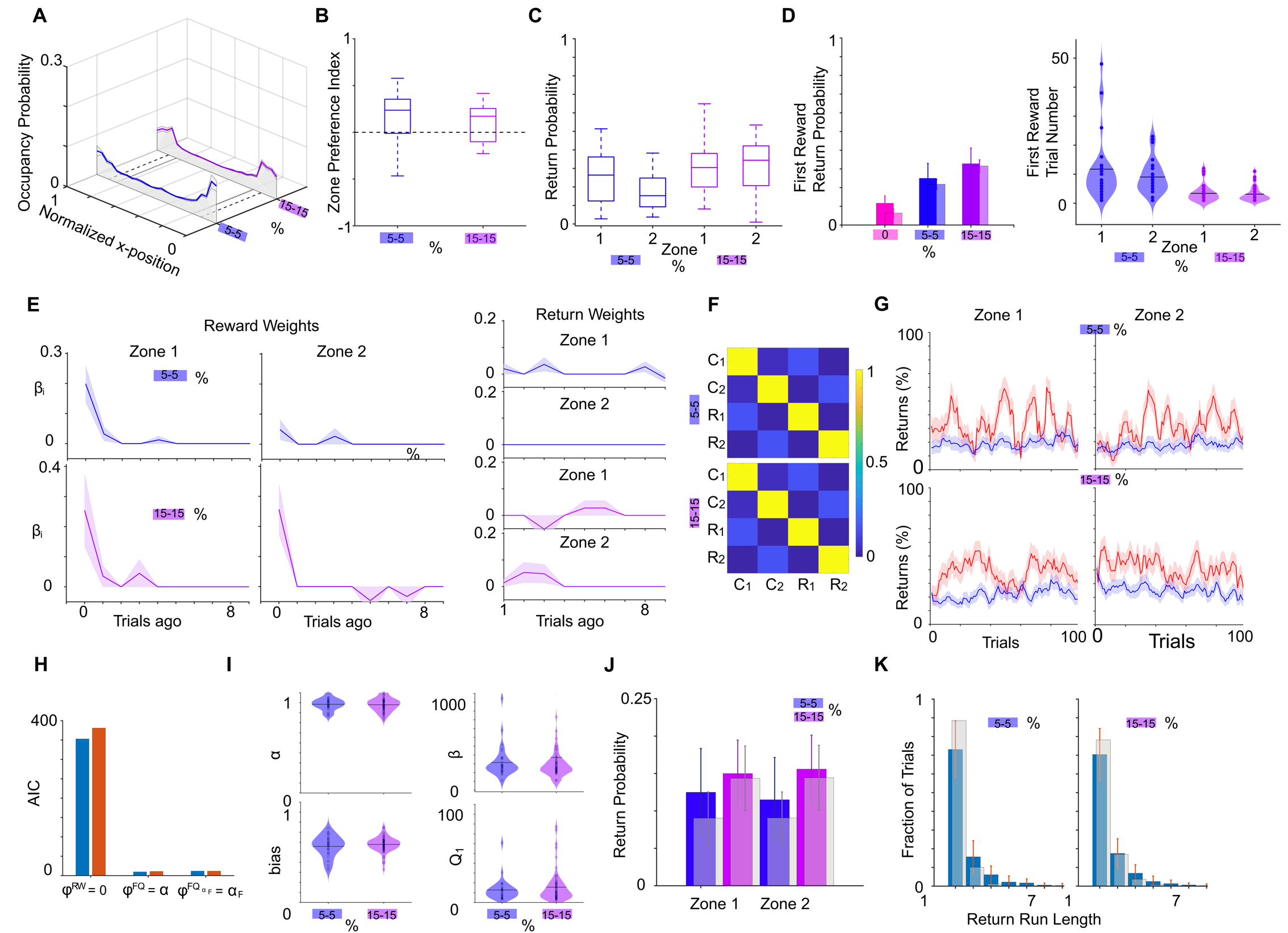

Supplement: S2 Fig — A Occupancy distribution for 5% and 15% double-sided stimulation data. B Preference index. C Return probability to zones 1 and 2 for both conditions. D Left: Return behavior on the first reward (to either zone) compared to the first trial return for unstimulated controls. Right: First rewarded trial number. E Left and center: Logistic regression weights of returns against the reward history for both population data to each zone independently. Right: Logistic regression weights for returns against return choice history. F Pearson correlation for rewards (R) and returns (C). G Return behavior as 5-trial moving average. Red curves: rewarded trials, blue curves: unrewarded trials to the same zone. H AIC score for the three RL models. I Best-fit parameter values of the FQ model. J Generative testing of the FQ model: comparison of the return probability (exp. data: colored, model: grey). K Generative testing of the FQ model: Return run lengths (exp. data: blue, model: grey). (PDF) [file pone.0239616.s002.pdf]

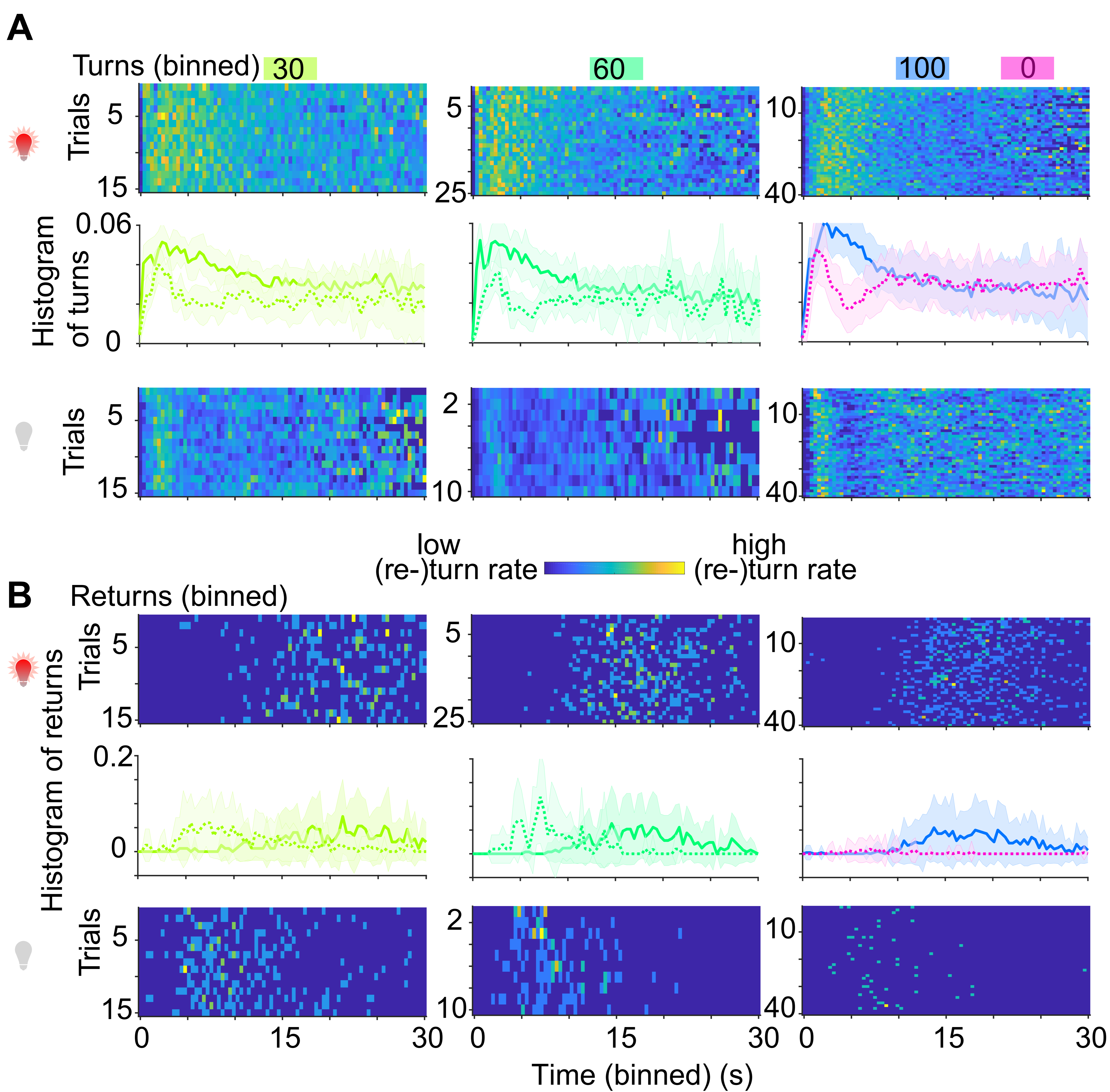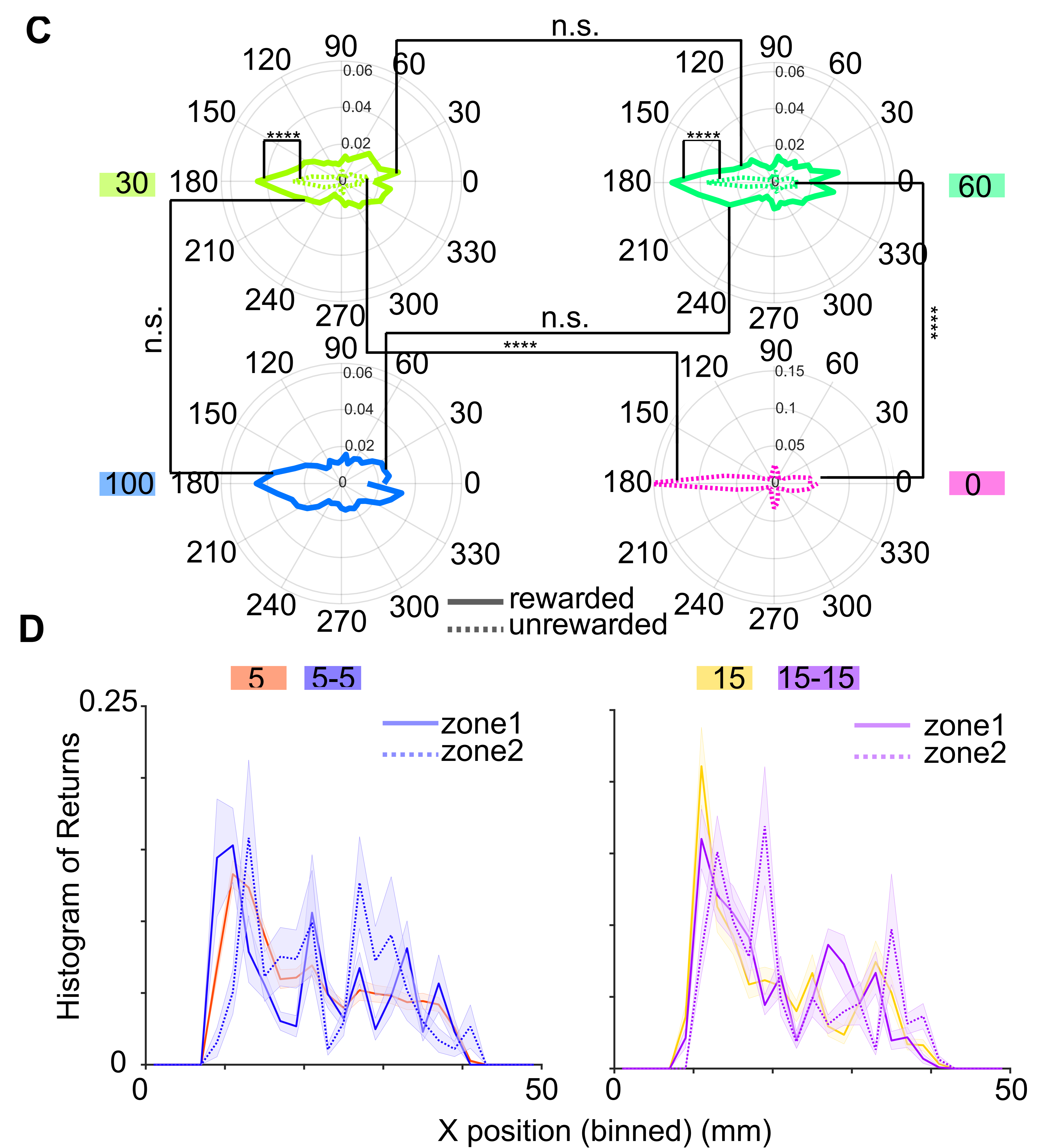

Supplement: S3 Fig — A Top row: Turns, as a proxy for local search, (binned) in time since trial start for 30, 60 and 100/0% conditions. Middle row: histogram of temporal turn distribution. Solid drawn curve corresponds to rewarded trials (top row) and dashed curve corresponds to unstimulated trials (bottom row). Bottom row: turns in time since trial start for unstimulated trials. B Top row: returns on rewarded trials in time since trial start for the same fly populations. Middle row: histogram of returns. Solid curve: stimulated returns, dashed curve: unstimulated returns. Bottom row: Unstimulated returns. C Polar plots of angular distributions of walking traces in the reset zone, for 30%, 60%, 100% populations and unstimulated controls (clockwise). Solid lines: rewarded trials, dashed lines: unrewarded trials. (****: p < 0.0001, two-way Kolmogorov-Smirnoff test.) D Comparison of return location (maximum position of a trial) for 5 and 15% single and double sided condition fly populations. Double sided cases have rewards in both zones and thus returns to both zones are separated. (PDF) [file pone.0239616.s003.pdf]

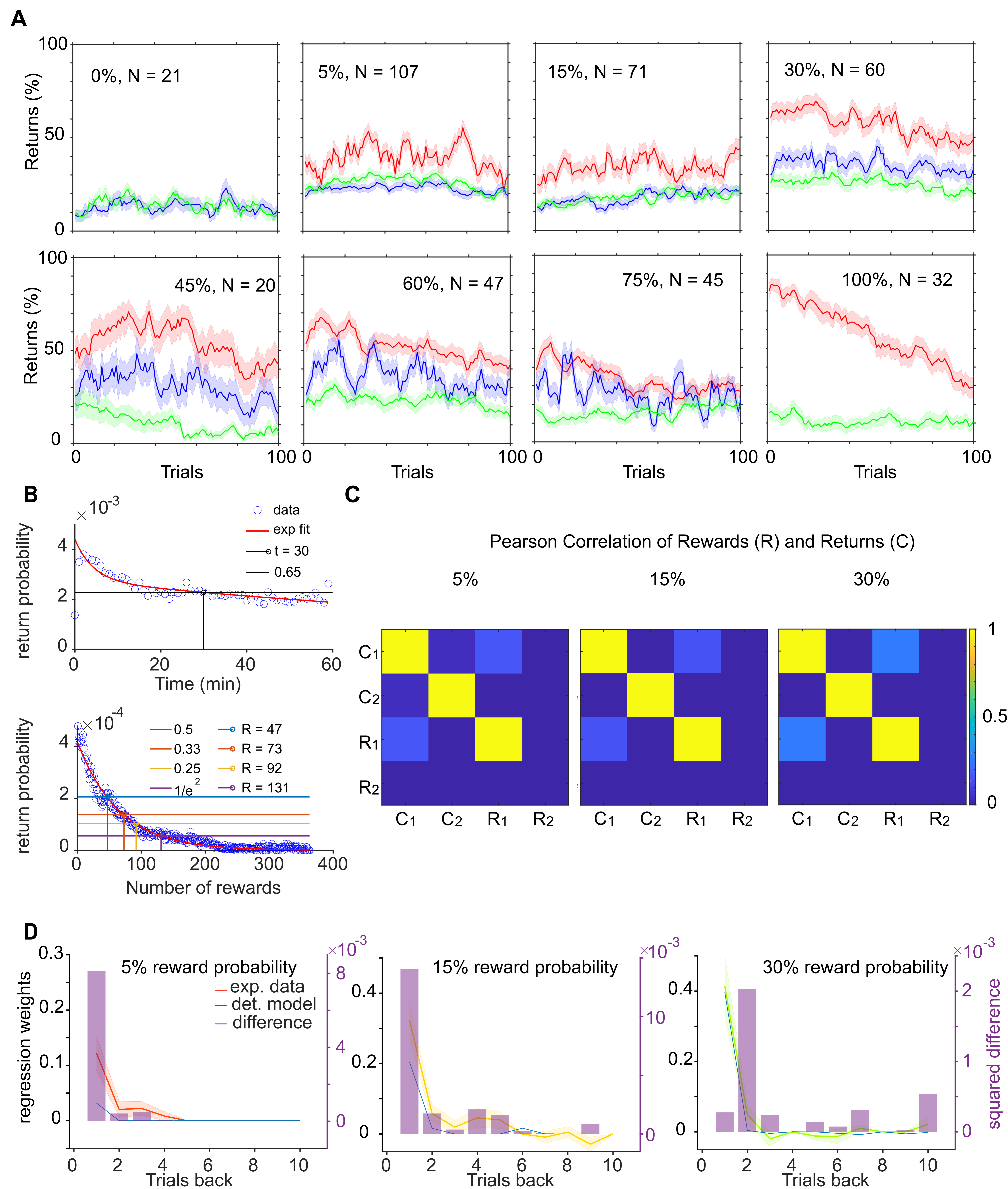

Supplement: S4 Fig — A 5-trial moving averages of the returns over trials for 0-100% stimulation probability conditions. Red curves show returns upon rewards, blue curves show returns to the rewarded zone without rewards and green curves show returns to the unstimulated zone.B Upper panel, return probability as a function of elapsed time from the start of the behavioral session. Single exponential fit (in red) to observer data. Blue circles indicate return probability of flies averaged across all reward probability conditions across time. At 30 min from the start of the behavioral session average probability of return drops by 35% (black intercept). Lower panel shows return probability averaged across all probability conditions as a function of number of rewards experienced by flies. Number of rewards and corresponding return probability are shown in legend C Pearson correlation of rewards and returns (choices) for 5,15 and 30% conditions. C Logistic regression of simulated data to rewards. Simulated data was generated with 50% return probability upon a reward. Curves show regression weights for different stimulation probabilities (5-30%). D The regression weights for experimentally observed data (color coded for each probability condition) from flies run on different probability conditions, deterministic model (blue curve) that responds to only immediate rewards with 0.5 return rate and difference between them (magenta bars). (PDF) [file pone.0239616.s004.pdf]

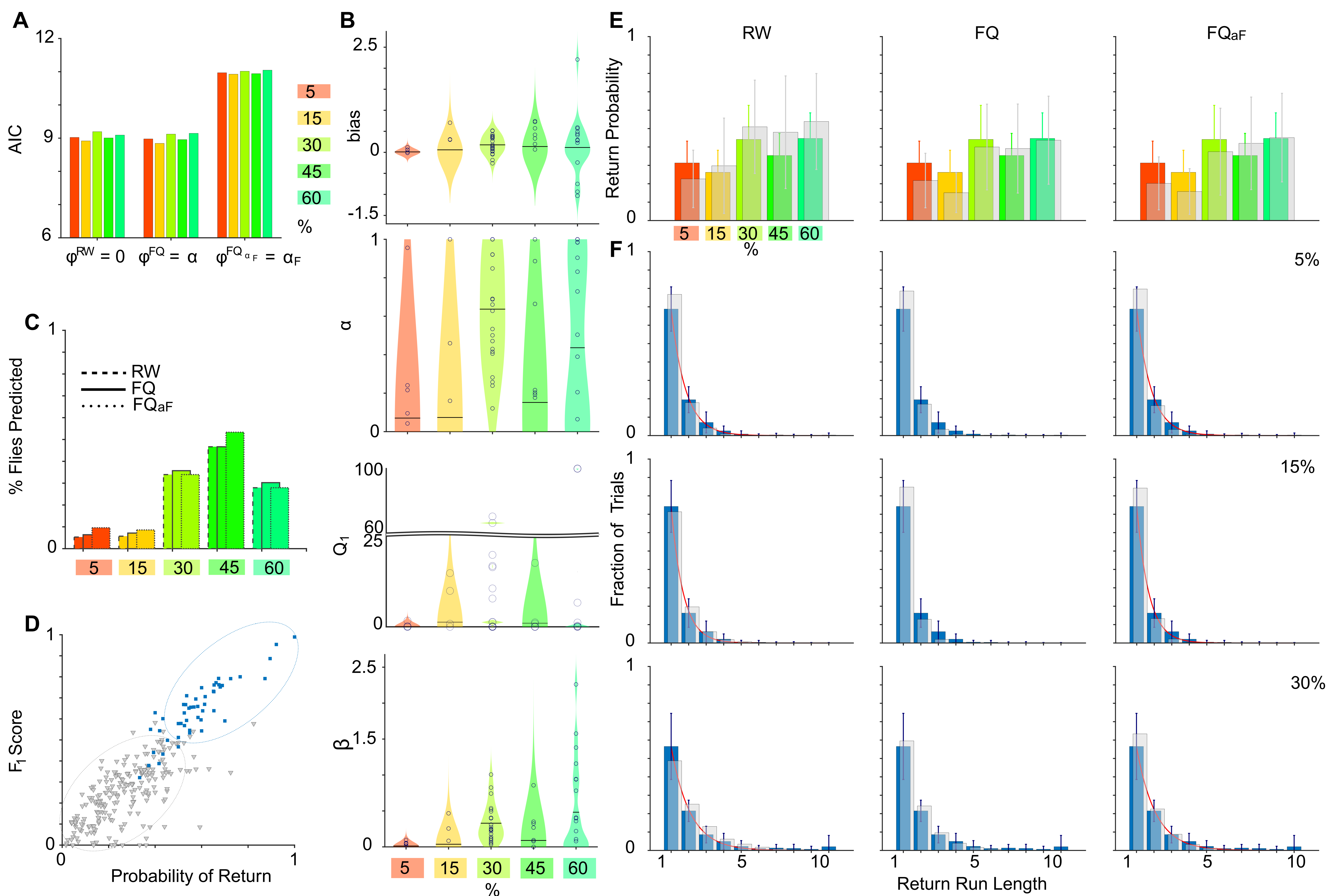

Supplement: S5 Fig — A AIC scores for the three RL models on 5-60% data. The lower the AIC score, the better the model captures the data while excessive parameters are punished. B Best-fit parameter values of the FQ model for each fly (circles) and population averages (solid lines in the violins). C Predictive test of the FQ model. Number of flies that could be predicted with more than 50% accuracy (F1 score) for each model. Total number of flies per condition: N5% = 94, N15% = 70, N30% = 56, N45% = 15, N60% = 45. D F1 score against data choice probability. If choices made up less than 50% of the data, the model had a poor predictive power. Dashed ellipses visualize clustering of the data with high and low F1 score. E Comparison of generative properties of the three RL models: Return probability. F Comparison of generative properties of the three RL models: Return run lengths. Red curves: exponential fits. (PDF) [file pone.0239616.s005.pdf]
