# Supplementary material for: Reward foraging task and model-based analysis reveal how fruit flies learn value of available options
Source: PLoS One. 2020 Oct 2;15(10):e0239616. doi: 10.1371/journal.pone.0239616 (PMC7531776; doi:10.1371/journal.pone.0239616)
Supplement: Attachment — Submitted filename: Response to Reviewers.docx [file pone.0239616.s007.docx]

**Rebuttal Letter**

*Reviewers Comments to the Author*

Reviewer #2: Fig. 1 D,G, and H still need labels to specify control genotypes. Other than that, the authors have addressed all of my concerns.

*Our response*

We addressed this issue and indicated clearly the control genotypes for Fig. 1D, G, and H. At the beginning of the Fig.1 legend, the second sentence, we also say what do we mean by gen.controls.
